# A systematic review of surgical margins utilized for removal of cutaneous mast cell tumors in dogs

**DOI:** 10.1186/s12917-019-2227-8

**Published:** 2020-01-06

**Authors:** Laura E. Selmic, Audrey Ruple

**Affiliations:** 1 Department of Veterinary Clinical Sciences, The Ohio State University College of Veterinary Medicine, 601 Vernon L Tharp St, Columbus, OH 43221 USA; 2 Department of Public Health, Purdue University, 725 Harrison Street, West Lafayette, IN 47906 USA

**Keywords:** Dog, Mast cell tumor, Surgical margin, Surgery

## Abstract

**Background:**

Traditionally, wide lateral surgical margins of 3 cm and one fascial plane deep have been recommended for resection of canine cutaneous mast cell tumor (MCT). Several studies have been published assessing surgical margins of less than this traditional recommendation. The objective of this systematic review was to determine if resection MCT with lateral surgical margins < 3 cm results in low rates of incomplete resection and local tumor recurrence. Systematic searches of digital bibliographic databases were performed with two authors (AR & LES) screening abstracts to identify relevant scientific articles. Studies regarding surgical treatment of dogs with cutaneous MCT were reviewed. Data abstraction was performed and the quality of individual studies and the strength of the body of evidence for utilization of surgical margins < 3 cm for removal of MCTs was assessed.

**Results:**

From the initial 78 citations identified through the database searches, four articles were retained for data abstraction after both relevance screenings were performed. Two studies were retrospective observational studies, one was a prospective case series and one was a prospective clinical trial. Assessment of the quality level of the body of evidence identified using the GRADE system was low. Excision of MCT at 2 cm and 3 cm was associated with comparably low rates of incomplete excision and recurrence.

**Conclusions:**

Despite the low quality of the overall body of evidence, a recommendation can be made that resection of canine cutaneous MCTs (< 4 cm) of Patnaik grade I and II with 2 cm lateral margins and 1 fascial plane deep results in low rates of incomplete excision and local tumor recurrence.

## Background

Mast cell tumor (MCT) is the most common malignant skin tumor in dogs [1–4]. The biological behavior of the tumor is often estimated by clinical staging results and histologic grading [5–12]. The standard of care for local mast cell disease is surgical excision with removal of margins of surrounding normal tissue; termed the surgical margins [13]. The aim of surgical treatment is to completely remove the tumor to minimize the chance of local tumor recurrence [13]. Most canine MCTs are not biopsied prior to excision due to risk of degranulation so histologic grade is often unknown at the time of surgery. Commonly the extent of gross disease is determined by manual palpation, which has very poor inter-observer agreement, and rarely by advanced imaging [14]. Microscopic mast cell disease can extend beyond palpable gross tumor margins, so traditionally, wide margins of 3 cm laterally and one fascial plane deep have been recommended for resection of all MCTs [15, 16].

Given that resection of these wide margins can cause significant patient morbidity, several investigators have assessed decreased lateral margins and the effect on completeness of excision or local tumor recurrence [17–19]. The ideal surgical margins to minimize recurrence and minimize patient morbidity have not yet been elucidated with a prospective randomized trial. To date, no systematic review has been performed to assess these studies and potential biases for practicing veterinarians. It is the authors’ hope that these systematic review findings could help inform practicing veterinary surgeons and help direct further research into this area. Thus, the objective of this systematic review was to determine if resection of canine cutaneous MCTs with lateral surgical margins < 3 cm results in low rates of incomplete resection and local tumor recurrence.

## Results

**Fig. 1:**
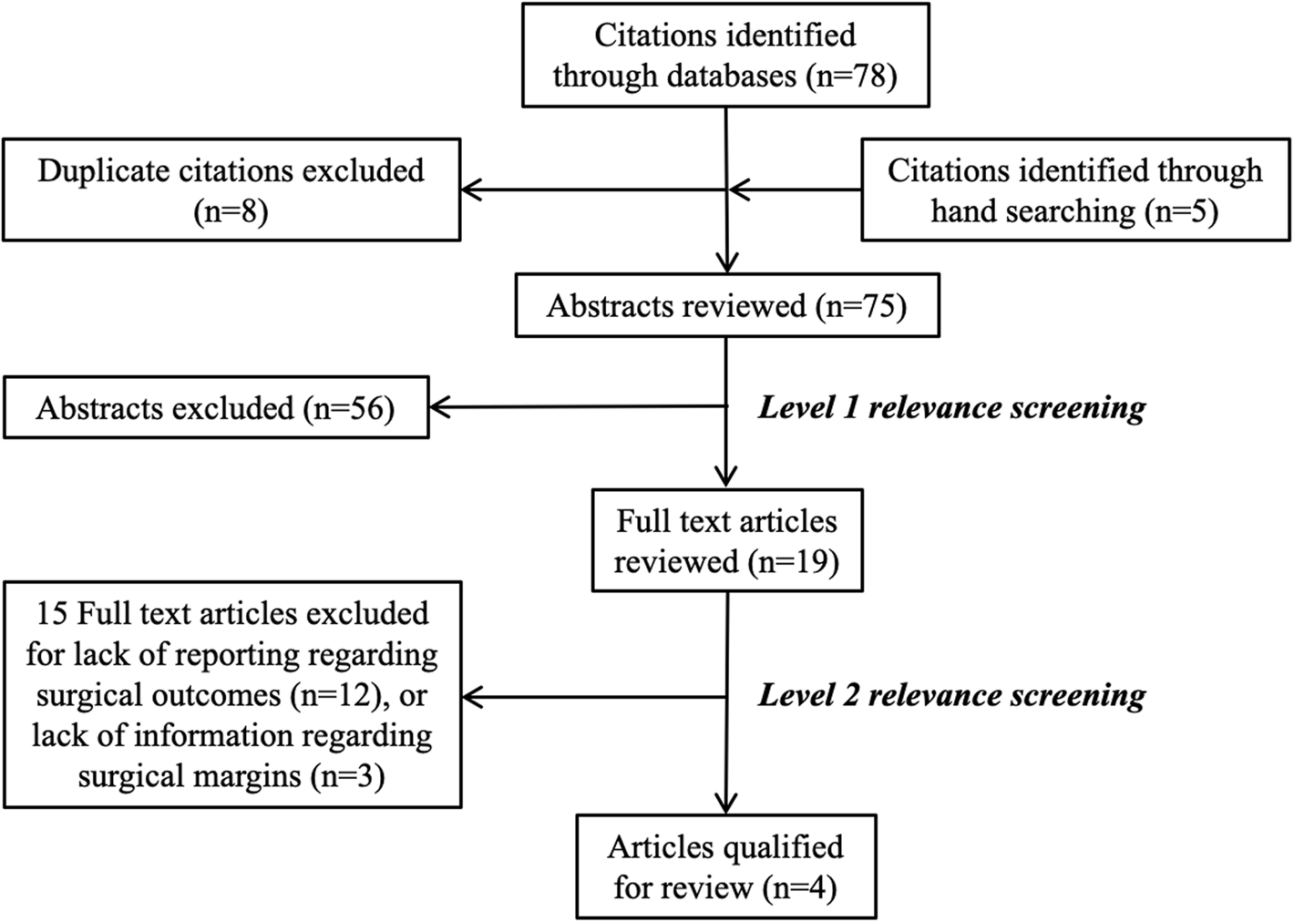
Flowchart showing numbers of manuscripts at each stage of the relevance screening process

The results of the literature search and relevance screening is summarized in Fig. 1. From 78 citations captured through database searches, only four articles remained after completion of both relevance screenings. The results of data collection are summarized in Table 1. These four articles addressed surgical outcome of MCT resection and included two retrospective case studies, one prospective case series, and one prospective clinical trial [16–19].

**Table 1:** Summary of data abstraction from the 4 manuscripts qualifying for review

First author	Fulcher	Pratschke	Seguin	Simpson
Year study published	2006	2013	2001	2004
Study description	Clinical trial	Case series	Case series	Case series
Prospective or retrospective	Prospective	Retrospective	Retrospective	Prospective
Tumor location	Cutaneous any site	Cutaneous and SQ	Cutaneous any site	Cutaneous any site
# dogs (MCTs)	16 (23 MCT)	40 (47 MCT)	55 (60 MCT)	21 (23 MCT)
Study groups	Single arm all had surgery (subgroup analysis where 1 cm margins were assessed also)	Single arm all had surgery	Single arm all had surgery and all PG 2	Single arm all had surgery (subgroup analysis where 1 cm and 2 cm margins were assessed also)
Kiupel grading	n/a	37 tumors low grade, 4 tumors high grade	n/a	n/a
# dogs with PG I with surgery	4	21	0	3
# dogs with PG II with surgery	19	18	55	20
# dogs with PG III with surgery	0	2	0	0
Surgical margins utilized	2 cm lateral, 1 fascial plane deep	Lateral margin proportional to size of tumor. (range 5 mm- 4 cm), minimum 1 well defined fascial plane deep	2-3 cm lateral margins and additional tissue deep to dogs that had incomplete resection of MCT by rDVM	3 cm lateral and 1 fascial plane deep.
Incomplete margin definition	Incomplete MCT at margins or within 1 mm of margins	Incomplete MCT at margins or within 1 mm of margins	Incomplete MCT at margins, complete but close within 1 mm of margins	Incomplete MCT at margins, complete but close within 1 mm of margins
Proportion with incomplete margins	2 cm: 10.5% (both PG2) 1 cm: 0% (PG1); 31.6% (PG2)	14.9%	1.7%	Total 1 cm: 21.7%, 2 cm: 0%, 3 cm 0% PG1: 1 cm: 0%, 2 cm: 0%, 3 cm: 0% PG2: 1 cm: 25.0%, 2 cm 0%, 3 cm 0%
Local tumor recurrence rate in dogs treated with surgery alone	0%	2.1% (PG3 tumor with complete margins)	5% (2 complete and 1 incomplete)	0%
Time to tumor recurrence (in cases that tumor recurred)	> 538 days	45 days*	61 days	> 603 days

*Represents value for one dog

*MCT* Mast cell tumor; *PG* Patnaik grade; *rDVM* Referring veterinarian; *SQ* Subcutaneous; *TFI* Tumor free interval

Using the GRADE system, the overall quality of evidence for this body of literature was low. This was because the initial quality of the body of evidence was rated as low based on the design of the included studies (most were observational and the clinical trial lacked randomization or a control group). Factors that could have increased the overall quality of the body of evidence, such as presence of a large effect size, a consistent dose response relationship, or evidence that plausible confounding, if present, would reduce the demonstrated effect. However, the low risk of bias, the consistency of results, the ability to directly compare outcomes, the lack of imprecision, and the absence of evidence to indicate publication bias has occurred did not lower the overall quality score. Thus, the body of available evidence by which we can assess outcomes related to incomplete excision and local recurrence were classified as low quality overall.

The individual manuscripts had between 16 and 55 dogs each, however some dogs had concurrent MCTs removed. In the studies, all experiments were single arm with no control group. All studies assessed cutaneous MCT at any location and only one study allowed inclusion of subcutaneous MCT [17]. Most tumors in the four studies were Patnaik (PG) I or II and only one of the studies evaluated tumors using the Kiupel grading system [17]. The two prospective studies utilized small sample sizes (16 and 21 dogs) [16, 18]. Given that only one study utilized the Kiupel grading system this was not used in our analysis.

All studies evaluated dogs with MCT < 6 cm and had surgery to treat MCT with different lateral surgical margins. Fulcher et al. utilized 2 cm lateral margins; Seguin et al. used 2-3 cm lateral margins; Simpson et al. used 3 cm lateral margins, and Pratschke et al. utilized lateral margins proportional to tumor size (5 mm to 4 cm) with the exception of one dog with a 6 cm tumor that received 4 cm lateral margins. Two studies further evaluated different lateral margins with assessment of smaller margins on the resected specimen in addition to the margin taken at surgery. Fulcher et al. assessed 1 cm lateral margins (in addition to 2 cm surgical margins) and Simpson et al. assessed 1 cm and 2 cm lateral margins (in addition to 3 cm surgical margins).

Considering all studies, the lateral surgical margins had variable ranges of incomplete histologic excision (3 cm: 0–1.7%, 2 cm: 0–10.5%, 1 cm: 0–31.6%). The study definition of incomplete excision is outlined in Table 1. The deep surgical margin was the same for all studies, with resection including one fascial plane. For the outcome of local tumor recurrence we only considered the margins that were measured on the patient and were used for resection of the tumor at surgery. Fulcher et al. used 2 cm lateral margins, and Simpson used 3 cm margins; and saw no recurrence with a median tumor free interval until recurrence as not reached at > 538 days. Only one dog had recurrence in the Pratschke et al. study at 45 days. In the Seguin et al. study, 3 tumors recurred at a median tumor free interval of 61 days (range: 51 to 252 days); two were completely excised originally and one was incompletely excised.

## Discussion

Our review of the current literature indicate there is evidence to suggest that the use of lateral surgical margins of < 3 cm for resection of PG I and II MCT less than 4 cm in size are associated with low rates of incomplete excision and local tumor recurrence. Utilizing the GRADE system, the body of evidence was determined to be low quality for surgical margins for MCT. Despite the overall GRADE quality rating of the evidence base, we can still conclude that surgical margins of 2 cm lateral and one fascial plane deep are associated with a low rate of incomplete excision of PG I and II canine MCT (< 4 cm) (0–10.5%) and low rates of recurrence (0%) given the current body of literature [16–19]. This is due in part to the fact that significant barriers exist to being able to perform studies with control groups and randomized design to evaluate surgical margins for malignant tumors. Specifically, it is difficult to accrue patients for these studies given the potential negative consequences of increased risk of local recurrence and associated morbidity for malignant tumors if incomplete excision occurs. Thus, it is not likely that a higher quality of evidence on this topic will be forthcoming.

The study populations in the four manuscripts included in this review consisted of dogs with MCT (< 6 cm) of PG I and II. Only one study included 2 PG III tumors and no studies included tumors larger than 6 cm diameter. Increased PG and increased tumor size are both factors that have been previously reported to be associated with more biologically aggressive disease. Large tumors and those with PG III typically have more extensive microscopic disease, which in turn makes them more difficult to completely excise and it is therefore expected they will have higher local recurrence rates [7, 20, 21]. The lack of study populations that included dogs with large tumors or those graded III on the Patnaik grading system prevent any conclusions being made in regards to surgical margins for those populations using the existing data. Thus, the recommendation for surgical margins determined from this systematic review cannot be extrapolated to larger tumors or those that are categorized as PG III. It is possible given the influence of size and histologic grade on completeness of histologic excision and local recurrence rates, and the differences in dog size that a single metric measurement may not be the most appropriate for surgical margin recommendations for MCT removal. Additionally, only one of the four included studies within this systematic review evaluated MCT using the Kiupel grading system, so conclusions cannot be directly made from this review regarding margin recommendations for tumors graded using this system. Further research is required to establish margin recommendations for more diverse patient populations with MCT.

A limitation of this review is that there were differences in the ways the included manuscripts assessed and classified surgical margins as being histologically incomplete. Two studies classified incomplete surgical margins as the presence of mast cells at the surgical margin and two classified margins as incomplete if mast cells were present within 1 mm of the surgical margin. The classification of margins as incomplete can be challenging with MCT due to inherent difficulties with differentiating between neoplastic mast cells and normal mast cells. Also, differences in the number of sections of the surgical margins evaluated may have influenced the number of margins determined to be incomplete. In the manuscripts included in this review, surgical margin assessment was described in detail in the Simpson et al. study with 4 full thickness sections assessed for 1 and 2 cm surgical margins and 8 sections assessed for 3 cm surgical margins. Similarly, the Fulcher et al. study used 4 sections for 1 cm surgical margins and 8 sections were evaluated for 2 cm surgical margins. In the other studies the number of sections assessed was not reported.

General limitations of this systematic review should be considered when interpreting the results. Despite an extensive search of multiple databases and identification of a high number of possible studies for inclusion only four published studies that fit inclusion criteria. In addition, the authors did not contact the corresponding author of manuscripts that did not contain details on surgical margins or outcomes to assess if these would also fit inclusion criteria. This may have led to exclusion of some studies where information may have been available. Conclusions made in this systematic review are based on only four papers. With the exclusion of papers not written in English we may have missed possible eligible manuscripts although this number is estimated to be low. It is possible that this review is susceptible to publication bias given only manuscripts published in peer reviewed journals were eligible for inclusion.

## Conclusion

There is evidence to suggest that most PG I and II MCTs that are smaller than 4 cm in size can be excised completely with 2 cm margins despite the fact that the overall quality of published evidence is classified as low. However, this review should encourage further study in this area, as use of smaller surgical margins can positively influence patient morbidity, owner satisfaction, and reduction in financial burden.

## Methods

### Overview

A review protocol based on PRISMA was designed prior to initiating this review (Additional file 1). The protocol was unable to be registered as no applicable veterinary registry existed at the time of writing and human registries will only permit registration of either human studies or animal studies related to human health. Systematic searches of digital bibliographic databases were performed in order to identify studies pertaining to treatment of dogs with cutaneous MCT. Two authors performed relevance screenings to identify which manuscripts should be included in the review. Data abstraction was performed and the quality of individual studies and the strength of the body of evidence for utilization of surgical margins < 3 cm for canine cutaneous MCTs was assessed.

### Literature search

Electronic literature searches were performed in PubMed (1950 to present), Web of Science (1900 to present), Medline (1950 to present), CAB Abstracts (1973 to present), conducted in June 2016. Search terms that described the population, intervention of surgical resection and comparison (< 3 cm vs. ≥ 3 cm) of lateral surgical margins were identified in the Medical Subject Headings (MeSH) database. The complete search string utilized was {[dog OR canine] AND [mast cell tumor* OR mastocytoma OR mastocytosis OR mast-cell sarcoma] AND [surgical margin OR incomplete margin OR dirty margin OR surgical resection OR surgical excision OR surgery OR biopsy] AND [recurrence OR local control OR neoplasm recurrence, local OR neoplasm, residual]}.

The citations retrieved from each search were stored in commercially available reference management software.^1^ Electronic and hand scanning of the resultant citation library containing the citations from all searches was performed to identify any duplicate citations. If duplicity or multiplicity of the same citation was present, only the most complete citation was retained. Reviewers performed hand searching of article reference lists as the review progressed to identify other potentially relevant citations. If a potentially relevant citation was identified by this method, it was manually added to the citation library on the reference management software.

### Relevance screenings

The citations recovered from the literature search process were screened to identify and remove citations that were not relevant to the review. Eligible studies were primary research studies (experimental or observational) published in English that reported outcomes of surgical treatment of cutaneous MCT in dogs. In addition, these studies needed to assess surgical margins < 3 cm. Studies that were case reports, review articles, not written in the English language, or those that did not report outcomes (histologic completeness of surgical margins and local recurrence) of surgical treatment or that had an incomplete description of surgical margins used were excluded from the review. The relevance screening was a two-stage process.

Stage 1 of relevance screening involved two reviewers (AR and LES) independently reviewing each abstract title. Citations proceeded to the second stage of review if both reviewers agreed the citation described primary research assessing the outcome of surgical treatment of cutaneous MCT or did not contain enough information to determine eligibility. When the two reviewers did not initially agree about a citation, a discussion was raised and consensus was determined. If the manuscripts met all inclusion criteria determined for stage 1 of relevance screening and the study was published in English, the manuscript could advance to the next stage of the review. Stage 2 of relevance screening involved evaluation of the full manuscript using the full inclusion criteria and was conducted independently by the same reviewers (AR and LES). Similarly, any disagreements were resolved through discussion and consensus between reviewers.

### Data abstraction and quality assessment

For manuscripts that passed through both stages of review, data were abstracted by one reviewer (LES). The results of the abstraction were assessed by a second reviewer (AR) to determine accuracy and completeness. The data abstracted from individual studies included the author list, years the study was performed and reported, study design, study population, sample size, institution where the study was performed, number of subjects treated with surgery in study, number of dogs with Patnaik histologic grades [5] (PG) 1, 2 and 3 or Kiupel low and high histologic grade [22], number of dogs with each grade treated with surgery, and the surgical margins utilized. Data were grouped by the surgical margin (< 3 cm and ≥ 3 cm margins) and study specific estimates of proportions of incomplete surgical resection and clinically detectable local recurrence at the surgical site (when treated with surgery alone) were abstracted when available. Incomplete resection was defined as determination on histopathologic assessment of surgical margins of the presence of mast cells extending to surgical margins. Clinically detected local recurrence was defined as a mass arising within 3 cm of the surgical site or scar. Confirmatory testing was not required for inclusion.

The individual study quality was determined based on multiple criteria including: 1) representativeness of study population; 2) selection of study participants; 3) data collection methods utilized; and 4) statistical and analytic methods used. For assessment of the quality of the entire body of evidence, guidelines developed by The Grading of Recommendations Assessment, Development, and Evaluation (GRADE) Working Group were used [23].

### Data synthesis

The data were described in the form of the resultant narrative review.

## Supplementary information

**Additional file 1.** PRISMA-P compliant systematic review protocol completed prior to initiation of the review.

## Data Availability

All data generated or analyzed during this study are included in the published article.

## Abbreviations

GRADE: Grading of Recommendations Assessment, Development, and Evaluation
MCT: Mast cell tumor
MeSH: Medical Subject Headings
PG: Patnaik grade

## Footnotes

1 Endnote™ online, Philadelphia, PA
