# Supplementary material for: A systematic review of surgical margins utilized for removal of cutaneous mast cell tumors in dogs
Source: BMC Vet Res. 2020 Jan 6;16:5. doi: 10.1186/s12917-019-2227-8 (PMC6945696; doi:10.1186/s12917-019-2227-8)
Supplement: Supplementary file 1 — Additional file 1. PRISMA-P compliant systematic review protocol completed prior to initiation of the review. [file 12917_2019_2227_MOESM1_ESM.docx]

**Supplemental file 1:**

PRISMA-P compliant systematic review protocol completed prior to initiation of the review

**SECTION 1: ADMINISTRATIVE INFORMATION**

**TITLE**

**Item 1a: Identification. Identify the report as a protocol of a systematic review**

A systematic review of surgical margins utilized for removal of cutaneous mast cell tumors in dogs

**Item 1b: If the protocol is for an update of a previous systematic review, identify as such**

This protocol is not updating a previous systematic review.

**REGISTRATION**

**Item 2: If registered, provide the name of the registry (such as PROSPERO) and registration number**

The protocol was not registered with a registry as it did not qualify for registration with a human registry as this study was not regarding humans or a review of animal studies directly related to human health. At the time of writing we were not aware of a veterinary registry that could register this study.

**AUTHORS**

**Item 3a: Contact information. Provide name, institutional affiliation, email address of all protocol authors; provide a physical mailing address of corresponding author.**

Laura E. Selmic, BVetMed (Hons), MPH. (Corresponding author)

Email: Selmic.1@osu.edu

Department of Veterinary Clinical Sciences,

The Ohio State University College of Veterinary Medicine,

601 Vernon L Tharp St.,

Columbus,

OH 43221.

Audrey Ruple, DVM, MS, PhD.

Email: aruple@purdue.edu

Department of Public Health,

Purdue University, IN.

**Item 3b: Contributions. Describe the contributions of protocol authors and identify the guarantor of the review.**

LES is guarantor of the review. LES and AR together developed the selection criteria, search strategy, risk of bias assessment strategy, data abstraction information, drafted and approved the manuscript.

**AMENDMENTS**

**If the protocol represents an amendment of a previously completed or published protocol, identify as such and list changes; otherwise, state plan for documenting important protocol amendments**

The protocol is not an amendment of a previously completed or published protocol. If protocol is amended following performing the systematic review, we will describe the amendment, the rationale for the amendment and give the date the amendment.

**SUPPORT**

**Item 5a: Sources. Indicate sources of financial or other support for the review.**

No financial or other support was utilized for this review.

**Item 5b. Sponsor. Provide name for the review funding and/or sponsor.**

The review has no sponsors.

**Item 5c. Describe roles of funder(s), sponsor(s), and/or institution(s), if any, in developing the protocol.**

N/A

**SECTION 2: INTRODUCTION**

**RATIONALE**

**Item 6: Describe the rationale for the review in the context of what is already known**

Mast cell tumor (MCT) is the most common malignant skin tumor in dogs.^1-4^ The biological behavior of the tumor is often estimated by clinical staging results and histologic grading.^5-12^ The standard of care for local mast cell disease is surgical excision with removal of margins of surrounding normal tissue; termed the surgical margins.^13^ The aim of surgical treatment is to completely remove the tumor to minimize the chance of local tumor recurrence.^13^ Most canine MCTs are not biopsied prior to excision due to risk of degranulation so histologic grade is often unknown at the time of surgery. Commonly the extent of gross disease is determined by manual palpation, which has very poor inter-observer agreement, and rarely by advanced imaging.^14^ Microscopic mast cell disease can extend beyond palpable gross tumor margins, so traditionally, wide margins of 3cm laterally and one fascial plane deep have been recommended for resection of all MCTs.^15,16^

Given that resection of these wide margins can cause significant patient morbidity, several investigators have assessed decreased lateral margins and the effect on completeness of excision or local tumor recurrence.^17-19^ The ideal surgical margins to minimize recurrence and minimize patient morbidity have not yet been elucidated with a prospective randomized trial. To date, no systematic review has been performed to assess these studies and potential biases for practicing veterinarians. It is the authors’ hope that these systematic review findings could help inform practicing veterinary surgeons and help direct further research into this area.

**OBJECTIVES**

**Item 7: Provide an explicit statement of the question(s) the review will address with reference to participants, interventions, comparators, and outcomes (PICO).**

The objective of this systematic review was to determine if resection of canine cutaneous MCTs with lateral surgical margins < 3cm results in low rates of incomplete resection and local tumor recurrence.

**SECTION 3: METHODS**

**ELIGIBILITY CRITERIA**

**Item 8: Specify the study characteristics (such as PICO, study design, setting, time frame) and report characteristics (such as years considered, language, publication status) to be used as criteria for eligibility for the review.**

Systematic searches of multiple digital bibliographic databases (from 1950 to present) were performed in order to identify studies pertaining to treatment of dogs with cutaneous MCT. Eligible studies were primary research studies (experimental or observational) published in English that reported outcomes of surgical treatment of cutaneous MCT in dogs. In addition, these studies needed to assess surgical margins < 3cm. Studies that were case reports, review articles, not written in the English language, or those that did not report outcomes (histologic completeness of surgical margins and local recurrence) of surgical treatment or that had an incomplete description of surgical margins used were excluded from the review.

**Item 9: Describe all intended information sources (such as electronic databases, contact with study authors, trial registers or other grey literature sources) with planned dates of coverage**

Electronic literature searches were performed in PubMed (1950 to present), Web of Science (1900 to present), Medline (1950 to present), CAB Abstracts (1973 to present), conducted in June 2016. Reviewers also performed hand searching of article reference lists as the review progressed to identify other potentially relevant citations. If a potentially relevant citation was identified by this method, it was manually added to the citation library on the reference management software.

**Item 10: Present draft of search strategy to be used for at least one electronic database, including planned limits, such that it could be repeated.**

Electronic literature searches were performed in PubMed (1950 to present), Web of Science (1900 to present), Medline (1950 to present), CAB Abstracts (1973 to present), conducted in June 2016. Search terms that described the population, intervention of surgical resection and comparison (<3cm vs. ≥ 3cm) of lateral surgical margins were identified in the Medical Subject Headings (MeSH) database. The complete search string utilized was {[dog OR canine] AND [mast cell tumor* OR mastocytoma OR mastocytosis OR mast-cell sarcoma] AND [surgical margin OR incomplete margin OR dirty margin OR surgical resection OR surgical excision OR surgery OR biopsy] AND [recurrence OR local control OR neoplasm recurrence, local OR neoplasm, residual]}.

**STUDY RECORDS**

**Data management**

**Item 11a: Describe the mechanism(s) that will be used to manage records and data throughout the review**

The citations retrieved from each search were stored in commercially available reference management software (Endnote^TM^ online, Philadelphia, PA.). Electronic and hand scanning of the resultant citation library containing the citations from all searches was performed to identify any duplicate citations. If duplicity or multiplicity of the same citation was present, only the most complete citation was retained. Reviewers performed hand searching of article reference lists as the review progressed to identify other potentially relevant citations. If a potentially relevant citation was identified by this method, it was manually added to the citation library on the reference management software.

**Selection process. Item 11b: State the process that will be used for selecting studies (such as two independent reviewers) through each phase of review (that is, screening, eligibility and inclusion in meta-analysis)**

The citations recovered from the literature search process were screened to identify and remove citations that were not relevant to the review. The relevance screening was a two-stage process. Stage 1 of relevance screening involved two reviewers (LES and AR) independently reviewing each abstract title. Citations proceeded to the second stage of review if both reviewers agreed the citation described primary research assessing the outcome of surgical treatment of cutaneous MCT or did not contain enough information to determine eligibility (based on previously stated criteria). When the two reviewers did not initially agree about a citation, a discussion was raised and consensus was determined. If the manuscripts met all inclusion criteria determined for stage 1 of relevance screening and the study was published in English, the manuscript could advance to the next stage of the review. Stage 2 of relevance screening involved evaluation of the full manuscript using the full inclusion criteria and was conducted independently by the same reviewers (LES and AR). Similarly, any disagreements were resolved through discussion and consensus between reviewers.

**Data collection process. Item 11c: Described planned method of extracting data from reports (such as piloting forms, done independently, in duplicate), any processes for obtaining and confirming data from investigators**

For manuscripts that passed through both stages of review, data were abstracted by one reviewer (LES). The results of the abstraction were assessed by a second reviewer (AR) to determine accuracy and completeness.

**Data items. Item 12: List and define all variables for which data will be sought (such as PICO items, funding sources), any pre-planned data assumptions and simplifications.**

The data abstracted from individual studies included the author list, years the study was performed and reported, study design, study population, sample size, institution where the study was performed, number of subjects treated with surgery in study, number of dogs with Patnaik histologic grades (PG) 1, 2 and 3 or Kiupel low and high histologic grade, number of dogs with each grade treated with surgery, and the surgical margins utilized. Data were grouped by the surgical margin (<3cm and ≥ 3cm margins) and study specific estimates of proportions of incomplete surgical resection and recurrence at the surgical site (when treated with surgery alone) were abstracted when available. Incomplete resection was defined as mast cells extending to surgical margins.

**OUTCOMES AND PRIORITIZATION**

**Item 13: List and define all outcomes for which data will be sought, including prioritization of main and additional outcomes, with rationale.**

The primary outcome variables that will be sought are incomplete surgical resection and local recurrence. Incomplete resection was defined as determination on histopathologic assessment of surgical margins of the presence of mast cells extending to surgical margins. Clinically detected local recurrence was defined as a mass arising within 3cm of the surgical site or scar. Confirmatory testing was not required for inclusion.

**RISK OF BIAS IN INDIVIDUAL STUDIES**

**Item 14: Describe anticipated methods for assessing risk of bias of individual studies, including whether this will be done at outcome or study level, or both; state how this information will be used in the data synthesis.**

The individual study quality was determined based on multiple criteria including: 1) representativeness of study population; 2) selection of study participants; 3) data collection methods utilized; and 4) statistical and analytic methods used. For assessment of the quality of the entire body of evidence, guidelines developed by The Grading of Recommendations Assessment, Development, and Evaluation (GRADE) Working Group were used.^20^

**DATA SYNTHESIS**

**Item 15a: Describe criteria under which study data will be quantitatively synthesized.**

The review planned was qualitative.

**Item 15b: If data are appropriate for quantitative synthesis, describe planned summary measures, methods of handling data and methods of combining data from studies, including any planned exploration of consistency (Such as I2, Kendall’s tau)**

N/A

**Item 15c: Describe any proposed additional analyses (such as sensitivity or subgroup analyses, meta-regression)**

N/A

**Item 15d: If quantitative synthesis is not appropriate describe the type of summary planned.**

A systematic narrative summary of the data will be provided. Information will be presented in both text and tables to explain the review findings for the included studies.

**META-BIAS(ES)**

**Item 16: Specify any planned assessment of meta-bias(es) (such as publication bias across studies, selective reporting within studies)**

There is no planned assessment of metabiases in the narrative systematic review. We anticipate most studies will share the same biases.

**CONFIDENCE IN CUMULATIVE EVIDENCE**

**Item 17: Describe how the strength of the body of evidence will be assessed (such as GRADE)**

For assessment of the quality of the entire body of evidence, guidelines developed by The Grading of Recommendations Assessment, Development, and Evaluation (GRADE) Working Group were used.^20^

**References:**

1. Bostock DE. Neoplasms of the skin and subcutaneous tissues in dogs and cats. *Br Vet J.* 1986;142(1):1-19.

2. Finnie JW, Bostock DE. Skin neoplasia in dogs. *Aust Vet J.* 1979;55(12):602-604.

3. Rothwell TL, Howlett CR, Middleton DJ, Griffiths DA, Duff BC. Skin neoplasms of dogs in Sydney. *Aust Vet J.* 1987;64(6):161-164.

4. Brodey RS. Canine and feline neoplasia. *Adv Vet Sci Comp Med.* 1970;14:309-354.

5. Patnaik AK, Ehler WJ, MacEwen EG. Canine cutaneous mast cell tumor: morphologic grading and survival time in 83 dogs. *Vet Pathol.* 1984;21(5):469-474.

6. Bostock DE. The prognosis following surgical removal of mastocytomas in dogs. *J Small Anim Pract.* 1973;14(1):27-41.

7. Murphy S, Sparkes AH, Smith KC, Blunden AS, Brearley MJ. Relationships between the histological grade of cutaneous mast cell tumours in dogs, their survival and the efficacy of surgical resection. *Vet Rec.* 2004;154(24):743-746.

8. Simoes JP, Schoning P, Butine M. Prognosis of canine mast cell tumors: a comparison of three methods. *Vet Pathol.* 1994;31(6):637-647.

9. Gerritsen RJ, Teske E, Kraus JS, Rutteman GR. Multi-agent chemotherapy for mast cell tumours in the dog. *Vet Q.* 1998;20(1):28-31.

10. Ayl RD, Couto CG, Hammer AS, Weisbrode S, Ericson JG, Mathes L. Correlation of DNA ploidy to tumor histologic grade, clinical variables, and survival in dogs with mast cell tumors. *Vet Pathol.* 1992;29(5):386-390.

11. Turrel JM, Kitchell BE, Miller LM, Theon A. Prognostic factors for radiation treatment of mast cell tumor in 85 dogs. *J Am Vet Med Assoc.* 1988;193(8):936-940.

12. Krick EL, Billings AP, Shofer FS, Watanabe S, Sorenmo KU. Cytological lymph node evaluation in dogs with mast cell tumours: association with grade and survival. *Vet Comp Oncol.* 2009;7(2):130-138.

13. London CA, Thamm DH. Mast Cell Tumors. In: Withrow SJ, Vail DM, Page RL, eds. *Withrow & MacEwen's Small Animal Clinical Oncology.* 5th ed. St. Louis, MO: Elsevier; 2013:335-355.

14. Ranganathan B, Milovancev M, Leeper H, Townsend KL, Bracha S, Curran K. Inter- and intra-rater reliability and agreement in determining subcutaneous tumour margins in dogs. *Vet Comp Oncol.* 2018;16(3):392-398.

15. Thamm D, Vail D. Mast cell tumors. In: Withrow S, MacEwen E, eds. *Small Animal Clinical Oncology.* 3rd ed. ed. Philadelphia: WB Saunders Co; 2001:261-282.

16. Simpson AM, Ludwig LL, Newman SJ, Bergman PJ, Hottinger HA, Patnaik AK. Evaluation of surgical margins required for complete excision of cutaneous mast cell tumors in dogs. *J Am Vet Med Assoc.* 2004;224(2):236-240.

17. Pratschke KM, Atherton MJ, Sillito JA, Lamm CG. Evaluation of a modified proportional margins approach for surgical resection of mast cell tumors in dogs: 40 cases (2008-2012). *J Am Vet Med Assoc.* 2013;243(10):1436-1441.

18. Fulcher RP, Ludwig LL, Bergman PJ, Newman SJ, Simpson AM, Patnaik AK. Evaluation of a two-centimeter lateral surgical margin for excision of grade I and grade II cutaneous mast cell tumors in dogs. *J Am Vet Med Assoc.* 2006;228(2):210-215.

19. Seguin B, Leibman NF, Bregazzi VS, et al. Clinical outcome of dogs with grade-II mast cell tumors treated with surgery alone: 55 cases (1996-1999). *J Am Vet Med Assoc.* 2001;218(7):1120-1123.

20. Balshem H, Helfand M, Schunemann HJ, et al. GRADE guidelines: 3. Rating the quality of evidence. *J Clin Epidemiol.* 2011;64(4):401-406.
